# Microbial diversity and metabolic networks in acid mine drainage habitats

**DOI:** 10.3389/fmicb.2015.00475

**Published:** 2015-05-29

**Authors:** Celia Méndez-García, Ana I. Peláez, Victoria Mesa, Jesús Sánchez, Olga V. Golyshina, Manuel Ferrer

**Affiliations:** ^1^Department of Functional Biology-IUBA, Universidad de OviedoOviedo, Spain; ^2^School of Biological Sciences, Bangor UniversityBangor, UK; ^3^Department of Applied Biocatalysis, Consejo Superior de Investigaciones Científicas, Institute of CatalysisMadrid, Spain

**Keywords:** acid mine drainage, *Archaea*, *Bacteria*, *Eukarya*, filterable fraction of archaea, metabolic network, metagenomics, metaproteomics

## Abstract

Acid mine drainage (AMD) emplacements are low-complexity natural systems. Low-pH conditions appear to be the main factor underlying the limited diversity of the microbial populations thriving in these environments, although temperature, ionic composition, total organic carbon, and dissolved oxygen are also considered to significantly influence their microbial life. This natural reduction in diversity driven by extreme conditions was reflected in several studies on the microbial populations inhabiting the various micro-environments present in such ecosystems. Early studies based on the physiology of the autochthonous microbiota and the growing success of *omics*-based methodologies have enabled a better understanding of microbial ecology and function in low-pH mine outflows; however, complementary *omics*-derived data should be included to completely describe their microbial ecology. Furthermore, recent updates on the distribution of eukaryotes and archaea recovered through sterile filtering (herein referred to as filterable fraction) in these environments demand their inclusion in the microbial characterization of AMD systems. In this review, we present a complete overview of the bacterial, archaeal (including filterable fraction), and eukaryotic diversity in these ecosystems, and include a thorough depiction of the metabolism and element cycling in AMD habitats. We also review different metabolic network structures at the organismal level, which is necessary to disentangle the role of each member of the AMD communities described thus far.

## 1. Introduction

Acid mine drainage (AMD) refers to the acid runoff originating in (generally) abandoned mining areas. The chemical reactions leading to AMD have been described extensively (Druschel et al., 2004; Schippers, 2004), and autochthonous chemolithotrophs primarily contribute to the acidification of mining-related leachates (Baker and Banfield, 2003; Johnson and Hallberg, 2003).

**Figure 1:**
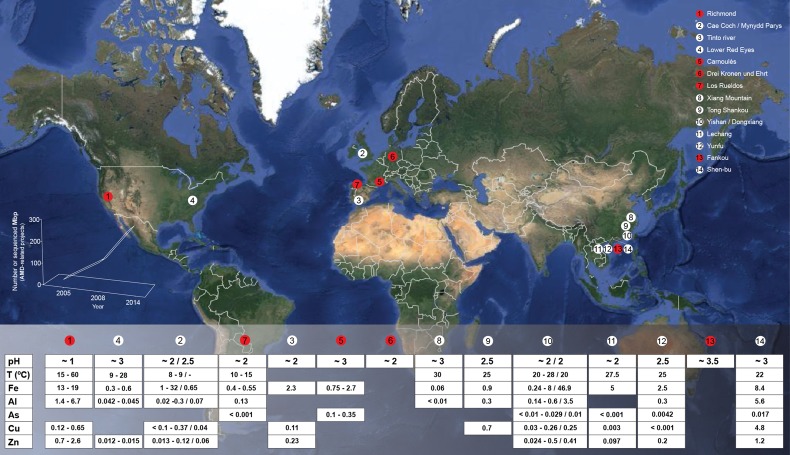
**Global map displaying the locations and geochemical features of a numerous set of characterized AMD emplacements**. Red circles represent that *omics*-based studies have been conducted in the indicated emplacement. Bottom-left chart reflects the evolution on the AMD-related New Generation Sequencing (NGS) data production in the period 2005–2014. Gaps in the table below correspond to the absence of available data. Some of recently described environments have not been included to facilitate readability, e.g., all the AMD emplacements described by Kuang et al. (2013) (in which metagenomic studies have been conducted as well).

AMD-related microbiota are present in niches without well-established boundaries. These microorganisms thrive in micro-environments including water, AMD bed sediments, and microbial macroscopic growths (streamers, mats, slimes, snottites or pipes, and drapes). Microbial macroscopic growth is present in ~30% of the AMD sites characterized globally (Mendez-Garcia et al., 2014) (Figure 1). Acid streamers are present in warm (>20°C) and greenish (high ferrous iron content), or cool (<15°C) and reddish (high ferric iron content) AMD solutions, while mats are irregularly spread among AMD sites when they are present. Additionally, the snottite microbial populations that have been characterized appear as stalactite-like growths in karst cave ceilings (Macalady et al., 2007; Jones et al., 2012) and mine roofs (pyrite, Ziegler et al., 2009, 2013; uranium, Zirnstein et al., 2012), and drapes exist in adits with low-temperature acidic runoffs (Johnson, 2012).

The primary environmental factors that shape AMD-associated microbial communities are pH, temperature, concentrations of dissolved metals and other solutes, total organic carbon (TOC), and dissolved oxygen (DO) (Figure 1). Such environmental factors, particularly pH (more than geographical location), are prime forces driving prokaryotic taxonomic beta-diversity variations among AMD sites (Tan et al., 2009; Kuang et al., 2013), whereas DO is the principal factor shaping the composition of the prokaryotic communities in AMD microbial growths, biofilms (Mueller et al., 2011; Mendez-Garcia et al., 2014), or snottites (Ziegler et al., 2013).

The diversity and microbiology in AMD systems have been thoroughly reviewed (e.g., Bond et al., 2000; Baker and Banfield, 2003; Johnson and Hallberg, 2003; Golyshina and Timmis, 2005; Johnson, 2007; Dopson and Johnson, 2012), and relevant metabolic properties, such as the capacity of microbes to live in acid conditions (e.g., Baker-Austin and Dopson, 2007), and their distinctive capabilities to metabolize carbon, iron, and sulfur, have been compared through environmental and genomic perspectives (Amaral Zettler et al., 2003; Johnson and Hallberg, 2008; Emerson et al., 2010; Bonnefoy and Holmes, 2012; Johnson et al., 2012). In contrast to previous reviews, herein we attempted to synthesize current knowledge regarding bacterial, archaeal and eukaryotic diversity in AMD habitats, and we included a thorough depiction of their global metabolism and element cycling patterns. We also reviewed different pathways and metabolic network structures at the organismal level, which is relevant to disentangle the role of each member of the different AMD communities described thus far. This review includes an overview of the microbial ecology in AMD systems inferred by culture- and community composition-based methodologies, as well as by integration of metagenomics, metatranscriptomics, metaproteomics, and metabolomics data.

## 2. Microbial diversity and associated metabolic capabilities in AMD systems

The observed microbial diversity at previously studied AMD sites includes organisms belonging primarily to the domains *Bacteria, Archaea* and, to a lesser extent, *Eukarya* (predominantly fungi and algae). Mining sites where AMD habitats have been extensively studied in terms of their microbial ecology include the Tinto River (SW Spain), the Richmond mine in Iron Mountain (CA, USA), Cae Coch (pyrite), and Mynydd Parys (copper) mines in UK, the Carnoulès (lead-zinc) mine in France, the Drei Kronen und Ehrt (pyrite) mine in Germany, and the Los Rueldos mercury mine in NW Spain. Others have been surveyed to generate a descriptive overview of their microbiology (see Figure 1 for details).

### 2.1. Bacterial diversity

**Figure 2:**
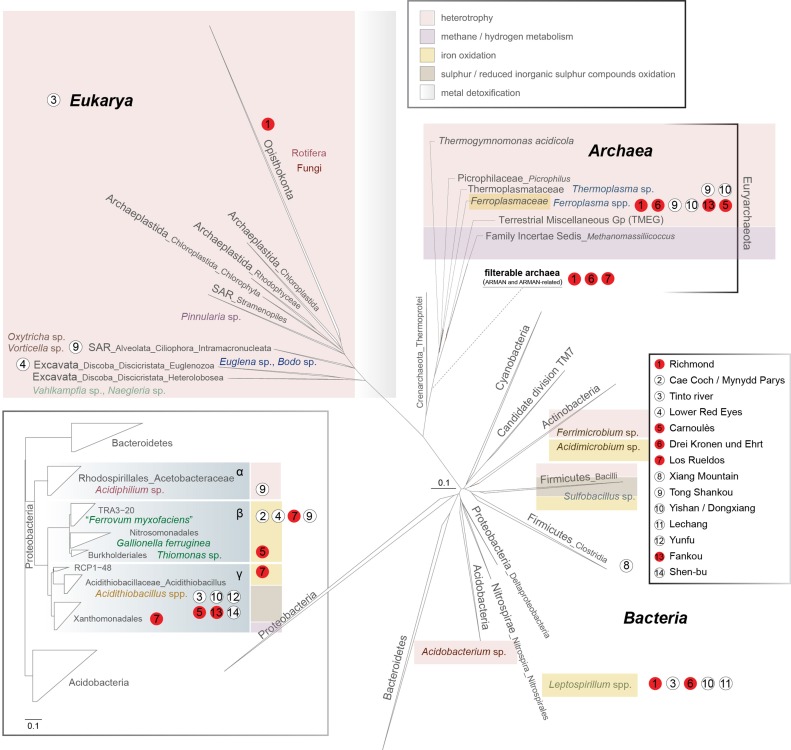
**16S rRNA gene-based phylogeny of the microbial domains in AMD systems** (***Bacteria**, **Archaea**, **Eukarya***). A sub-phylogeny for *Proteobacteria* is detailed in the bottom-left box (concerning the order *Acidithiobacillales*, please note that Williams and Kelly (2013) have proposed the existence of Acidithiobacillia classis nov. using complete multigenome/multiprotein alignment studies). The main ecological features of each group are displayed by a color code (top-right inset). Scale bars represent changes per site. The positioning of the archaea from the filterable fraction is tentative due to the lack of information referring to the group.

*Bacteria* inhabiting acidic waters, sediments and macroscopic growths associated with AMD systems belong primarily to the phyla *Proteobacteria, Nitrospirae, Actinobacteria, Firmicutes*, and *Acidobacteria*; other phyla, such as the *Bacteroidetes*, or the candidate division TM7, have been detected anecdotally in these environments (Figure 2).

*Proteobacteria* are widely distributed in acidic ecosystems (Figure 2). Amongst the most common genera that inhabit AMD environments, *Acidithiobacillus* spp. (Acidithiobacillia classis nov., Williams and Kelly, 2013) are acidophiles (optimal growth at pH 2–3) and exhibit mesophilic growth optima. These bacteria possess chemolithotrophic metabolism, by which they are able to oxidize Fe^2+^ and sulfur compounds (*At. ferrooxidans, At. ferridurans*, and *At. ferrivorans*), or just sulfur compounds (*At. caldus, At. thiooxidans*, and *At. albertensis*), under oxic conditions. *At. ferrooxidans* can perform CO_2_ and atmospheric nitrogen fixation (Valdes et al., 2008) (Table 1). *At. caldus* SM-1 fixes CO_2_ via the Calvin-Benson-Bassham (CBB) cycle, has an incomplete tricarboxylic acid cycle (TCA), and is also able to assimilate carbohydrates (You et al., 2011) (Table 1), but it has not yet been demonstrated that it can perform atmospheric nitrogen fixation. The *At. ferrivorans* draft genome exhibits genes potentially encoding CO_2_ fixation via the CBB cycle, and displays a complete repertoire of genes for nitrogen metabolism (Liljeqvist et al., 2011) (Table 1). *At. thiooxidans* is an extremely acidophilic, chemolithoautotrophic bacterium that obtains energy from the oxidation of sulfur and reduced inorganic sulfur compounds. Its draft genome possesses complete sets of genes for CO_2_ fixation via the CBB cycle (Levican et al., 2008), and for central carbon metabolism, but it lacks genes encoding 2-oxoglutarate dehydrogenase, which is involved in the TCA cycle, a characteristic genome signature for obligate autotrophs (Valdes et al., 2011) (Table 1).

**Table 1:** **Relation of available genome sequences for AMD-related microorganisms with cultured representatives**.

**Species**	**NCBI Reference**	**Status assembly**	**Related reference**
*Acidithiobacillus thiooxidans* ATCC 19377	NZ AFOH00000000.1	Scaffold	Levican et al., 2008
*At. feroooxidans*	NC 011206.1	Complete genome	Valdes et al., 2008
*At. caldus* SM-1	NC 015850.1	Complete genome	Valdes et al., 2009
*At. ferrivorans* SS3	NC 015942.1	Complete genome	Liljeqvist et al., 2011
*“Ferrovum myxofaciens*” P3G	NZ JPOQ00000000.1	Contig	Moya-Beltran et al., 2014
*Leptospirillum ferrooxidans* C2-3	NC 017094.1	Complete genome	Fujimura et al., 2012
*L. ferriphilum* ML-04	NC 018649.1	Complete genome	Mi et al., 2011
*Ferrimicrobium acidiphilum* DSM 19497	NZ JQKF00000000.1	Scaffold
*Acidimicrobium ferrooxidans* ICP	NC 013124.1	Complete genome	Clum et al., 2009
*Sulfobacillus acidophilus* TPY	NC 015757.1	Complete genome	Li et al., 2011
*Sulfobacillus acidophilus* NAL^T^	–	Complete genome	Anderson et al., 2012
*Sulfobacillus thermosuphidooxidans* str. Cutipay	NZ ALWJ00000000.1	Scaffold	Travisany et al., 2012
*Sulfobacillus thermosuphidooxidans* DSM 9293	PRJNA61271	Complete genome	–
*Alicyclobacillus acidocaldarius* subsp. *acidocaldarius* DSM 446	NC 013205.1	Complete genome	Mavromatis et al., 2010
*“Ferroplasma acidarmanus*” fer1	NC 021592.1	Complete genome	Allen et al., 2007
*Thermoplasma acidophilum* DSM 1728	NC 002578.1	Complete genome	Ruepp et al., 2000

*Betaproteobacteria* without specific taxonomic affiliations are present predominantly in less-restrictive pH and temperature conditions. The iron-oxidizing betaproteobacterium “*Ferrovum myxofaciens*” produces large amounts of exo-polysaccharides (Johnson et al., 2014) and is proposed to be the predominant member in streamer-type macroscopic growths at two mine sites in North Wales (Hallberg et al., 2006; Kimura et al., 2011), and in the Königstein uranium mine snottites (Brockmann et al., 2010). The “*Fv. myxofaciens*” P3G draft genome includes coding sequences for complete glycolysis and the TCA cycle, suggesting that it may be a facultative heterotroph. The presence of the full set of *nif* genes advances the notion that this bacterium may be able to fix atmospheric nitrogen (Moya-Beltran et al., 2014) (Table 1). The RuBisCO and phosphoribulokinase (*prkB*) genes for carbon fixation were observed to be highly expressed in a naturally occurring population of “*Ferrovum*” spp., while no nitrogen fixation-associated genes were detected; however, the expression of genes related to urea breakdown and nitrate reduction suggests the utilization of alternative nitrogen resources (Hua et al., 2014). Some *Betaproteobacteria* found in the Carnoulès lead-zinc mine are closely related to the neutrophilic iron oxidizer *Gallionella ferruginea* (Bruneel et al., 2006; Bertin et al., 2011). *Gallionella*-related iron oxidizers have been also extensively observed in metal-contaminated creeks in the former uranium-mining district of Ronneburg, Germany (Fabisch et al., 2013), as well as in the Yunfu sulfide mine, China (He et al., 2007). Others include putative heterotrophic growers, such as *Thiomonas* sp. (Storwartz, Ynysarwed and Parys Mountain mines), *Ralstonia* sp. (Tinto River, Shen-bu mine), and *Acidovorax* sp. (Tong Shankou and Yinshan mines) (Xie et al., 2007; Yin et al., 2008). *Thiomonas* spp. are facultative chemolithoautotrophs that grow optimally in mixotrophic media containing reduced inorganic sulfur compounds and organic supplements (Kelly et al., 2007; Arsene-Ploetze et al., 2010; Slyemi et al., 2011). The genus *Acidiphilium* (iron-reducing alphaproteobacterium) (Figure 2) appears frequently in AMD environments. *A. acidophilum* might thrive heterotrophically with *Acidithiobacillus ferrooxidans* and promote its growth (Liu et al., 2011). Heterotrophs in AMD systems create suitable environments for the growth of iron-oxidizers by removing organic components (lysates, exudates) that can be toxic to the primary producers (Bacelar-Nicolau and Johnson, 1999). For instance, heterotrophic *Acidocella* spp. (mesophilic alphaproteobacteria) are also found in acid mine waters (King and Parys copper mines and the Wheal Jane tin mine). *Acidisphaera* spp., which are aerobic heterotrophic alphaproteobacteria, have been detected in Japan and in the Roeros mining area in Norway (Hiraishi et al., 2000; Johnson et al., 2001); the latter AMD site is also inhabited by *Frateuria* spp. (*Gammaproteobacteria*, order *Xanthomonodales*). Among *Proteobacteria*, both *Sphingomonas*-like alphaproteobacteria (order *Sphingomonodales*) and *Ralstonia*-like betaproteobacteria (order *Burkholderiales*) are present in the Tinto River and Cae Coch mines.

Within the phylum *Nitrospirae*, the most relevant genus known to inhabit AMD systems is *Leptospirillum* (order *Nitrospirales*) (Figure 2). *Leptospirillum* spp. are chemolithoautotrophs that obtain energy from the oxidation of ferrous iron. *Leptospirillum* “group I” (*L. ferrooxidans*-related) and “group II” (*L. ferriphilum* and “*L. rubarum*”-related) have optimum growth temperatures of 26–30°C and 30–40°C, respectively. “*L. ferrodiazotrophum*” (“group III”) is proposed to be responsible for nitrogen fixation in AMD at pH levels below 1.0 in the Iron Mountain mine (Tyson et al., 2005; Goltsman et al., 2009). A phylogenetically distinct and “*L. ferrodiazotrophum*”-related putative new group of *Leptospirillum* spp. (“group IV”) has recently been detected via metagenomic data as a minority member in archaea-dominated low-pH biofilms in the Richmond mine (Goltsman et al., 2013). Other *Nitrospirae*-related groups have been recently obtained from the transcriptome of natural acid mine drainage biofilms collected at the Richmond mine, which include sequences related to the *Magnetobacterium* genus, as well as other uncultured and unclassified *Nitrospiraceae* (Aliaga Goltsman et al., 2014).

Iron-oxidizing, heterotrophic *Actinobacteria* (*Ferrimicrobium* spp. and *Acidimicrobium* spp.) are microorganisms that commonly thrive in these environments (Bond et al., 2000) (Figure 2). *Ferrimicrobium acidiphilum* can remove dissolved organic carbon, which is inhibitory to co-existing autotrophs, allowing their development in such systems (Bacelar-Nicolau and Johnson, 1999).

The ubiquitous *Acidobacteria* (mainly found in soils) thrive in relatively moderate acidic mine drainage-impacted environments (Figure 2). The few isolates belonging to this phylum that have been analyzed are primarily heterotrophs. Phototrophy and strict anaerobiosis also occur in this group (Ward et al., 2009). The most common genus present in AMD systems is the mesophilic (temperature growth range from 2 to 42°C, optimum of 30–35°C), obligate heterotroph *Acidobacterium*.

*Sulfobacillus* spp. (phylum *Firmicutes*, order *Clostridiales*) appear to thrive preferentially in warm AMD solutions (optimum growth at temperatures ~45°C) (Figure 2). *Sulfobacillus acidophilus* type strain NAL^T^ can grow autotrophically by oxidizing Fe^+2^, sulfur, or mineral sulfides, or heterotrophically on yeast extract (Anderson et al., 2012) (Table 1). *Sulfobacillus thermosulfidooxidans* (Golovacheva and Karavaiko, 1978) strain Cutipay is a mixotrophic, acidophilic, moderately thermophilic bacterium recently isolated from mining environments in the north of Chile (Travisany et al., 2012) (Table 1). Furthermore, draft genomes of the putative *Sb. thermosulfidooxidans* strain AMDSBA5 and *Sb. benefaciens* strain AMDSBA1, as well as from other 3 additional presumptively mixotrophic *Sulfobacillus* species with no cultured representatives, have been recently reconstructed from cultivation-independent sequencing of biofilms sampled from the Richmond mine (Justice et al., 2014). Additionally, firmicutes of the order *Bacillales*, family *Alicyclobacillaceae* (heterotrophs), have been detected in the Iron Mountain mine (Baker and Banfield, 2003). A ferrous iron-oxidizing bacterium classified under the genus *Alicyclobacillus* was also isolated from an enrichment culture obtained from the Matsuo mine acid drainage treatment plant (Japan) (Joe et al., 2007).

Some bacteria classified as affiliates of the candidate division TM7 have been detected in waste ore samples (pH 3.0) collected at an AMD site in the Anhui province of China (Hao et al., 2007), in macroscopic growths at the Los Rueldos abandoned mercury mine in NW Spain (Mendez-Garcia et al., 2014), and other AMD environments (Kuang et al., 2013) (Figure 2).

Sulfate-reducing bacteria (SRB) are chemoorganotrophic or chemolithotrophic organisms that use sulfate as a terminal electron acceptor and constitute a physiologically unique group of microorganisms that couple anaerobic electron transport to ATP synthesis (Barton and Fauque, 2009). The limited presence of these moderately acidophilic bacteria, with pH optimum ≥5, in AMD solutions, could be due to the high acidity and metal concentrations inherent to these environments (Cabrera et al., 2006). Acidophilic or acid-tolerant SRB inhabiting AMD-impacted sites have been isolated from acidic mine waters and sediments (Rowe et al., 2007; Sanchez-Andrea et al., 2012; Giloteaux et al., 2013), a salt marsh impacted by long-term AMD (Moreau et al., 2010), and sulphidic mine tailings (Wielinga et al., 1999; Fortin et al., 2000; Bruneel et al., 2006; Diaby et al., 2007). SRB may belong to the *Deltaproteobacteria, Firmicutes, Nitrospirae, Thermodesulfobacteria* and some archaeal taxa, but their presence at AMD sites is restricted to *Deltaproteobacteria*, also capable of iron reduction (Bond et al., 2000; Baker and Banfield, 2003), and *Firmicutes*.

### 2.2. Archaeal diversity

Archaea populating AMD sites generally belong to the order *Thermoplasmatales*, which, as per our current knowledge state, consists mostly of cell wall-lacking organisms with a single membrane bounding the cytoplasm and pleomorphic cell shapes. The most abundant genus of this order in AMD systems is *Ferroplasma*, which comprises iron-oxidizing hyper-acidophiles with optimum growth pH and temperature values of 1.2–1.7 and ~40°C, respectively (Edwards et al., 2000; Golyshina et al., 2000; Dopson et al., 2004; Golyshina and Timmis, 2005; Golyshina, 2011) (Figure 2). *Fp. acidiphilum* strain Y was reported to contain iron-protein-dominated cellular organization (Ferrer et al., 2007, 2008). Metagenome sequences derived from the Richmond mine AMD are available for *Ferroplasma* spp.: a genome from the isolate “*Fp. acidarmanus*” fer1 (1.94 Mbp, Allen et al., 2007) (Table 1) and metagenomic partial sequences from uncultured “*Ferroplasma*” type II (1.48 Mbp, Tyson et al., 2004) (Table 2). Baker and Banfield (2003) also detected a wide set of unculturable organisms related to *Thermoplasmatales* clones in these environments; these archaea (the so-called “alphabet plasmas”) (Table 2), which appear to be adapted to high biomass, metal-rich, low pH, 30–50°C habitats, are predicted to be facultative anaerobic heterotrophs, likely differing in their genetic capabilities for biosynthesis, motility and iron oxidation (Yelton et al., 2013).

**Table 2:** **Relation of NCBI available metagenome-derived genome reconstructions recovered from AMD systems**.

**NCBI project ID**	**Metagenome project**	**Reference**
PRJNA13696	AMD biofilm (Richmond mine)	Tyson et al., 2004
PRJNA29591	*Leptospirillum* sp. Group II	
PRJNA29593	*Leptospirillum* sp. Group III	
PRJNA29595	“*Ferroplasma acidarmanus*” Type I	Allen et al., 2007
PRJNA29597	*Ferroplasma* sp. Type II	
PRJNA29599	*Thermoplasmatales* archaeon GpI (G-plasma)	Tyson et al., 2004
PRJNA18537	AMD biofilm (Richmond mine)	
PRJNA18795	“*Leptospirillum rubarum*”	Lo et al., 2007
PRJNA37907	*“Leptospirillum ferrodiazotrophum*”	
PRJNA20823	AMD biofilm (Richmond mine)	
PRJNA38565	*Candidatus* Micrarchaeum acidiphilum ARMAN-2	Baker et al., 2010
PRJNA63555	*Candidatus* Parvarchaeum acidiphilum ARMAN-4	Baker et al., 2010
PRJNA63557	*Candidatus* Parvarchaeum acidiphilum ARMAN-5	Baker et al., 2010
PRJNA176861	*Leptospirillum* sp. Group IV	Goltsman et al., 2013
PRJNA40089	*Thermoplasmatales* archaeon A-plasma	Yelton et al., 2011
PRJNA40091	*Thermoplasmatales* archaeon E-plasma	Yelton et al., 2011
PRJNA40093	*Thermoplasmatales* archaeon I-plasma	Yelton et al., 2011
ACXK00000000	*Thermoplasmatales* archaeon C-plasma	Yelton et al., 2013
ACXK00000000	*Thermoplasmatales* archaeon D-plasma	Yelton et al., 2013
PRJNA184676	AMD (Kristineberg mine)	Auld et al., 2013
PRJNA248250	AMDs from metalliferous mining wastes in South China	
PRJNA193663	Stratified AMD streamer (Los rueldos)	Mendez-Garcia et al., 2014
PRJNA193664	Stratified AMD streamer (Los rueldos)	Mendez-Garcia et al., 2014
PRJNA193665	AMD mat (Los rueldos)	Mendez-Garcia et al., 2014

Various lineages of filterable archaea (the so-called “Archaeal Richmond Mine Acidophilic Nano-organisms,” ARMAN) have been discovered in AMD biofilms developing within the Richmond mine (Baker et al., 2006, 2010; Comolli et al., 2009) (Figure 2). ARMAN cells exhibit cytoplasmic volumes proposed to approach the minimum required for a free-living, independent life-style (Comolli et al., 2009). Five putative types of ARMAN have been detected in Iron Mountain: ARMAN-1 (Baker et al., 2006), ARMAN-2 (*Candidatus* Micrarchaeum acidophilum, Baker et al., 2006, 2010), ARMAN-3 (Baker et al., 2006), ARMAN-4 (*Candidatus* Parvarchaeum acidophilum, Baker et al., 2010) and ARMAN-5 (*Candidatus* Parvarchaeum acidophilus, Baker et al., 2010) (Table 2). Based on metagenomics analysis, types 2, 4, and 5 have average genome sizes of ~1 Mb. No isolates of filterable archaea have been cultured. The genomic features of ARMAN and imaging-based evidence for a possible physical relationship between archaea from the order *Thermoplasmatales* and ARMAN call into question the existence of an independent life-style. Additional archaeal (ARMAN-HM 2, ARMAN-HM 4, ARMAN-HM 7, and ARMAN-HM 29) 16S rRNA sequences from an acidic biofilm collected at the Harz-Mountains (Drei Kronen und Ehrt pyrite mine, Germany) have been deposited in public sequence databases (Ziegler et al., 2013). Related microorganisms (maximum 96% BLAST homology) have been detected in other acidic environments, including hot springs and pools, a boreal fen, the Tinto River water column and other AMD biofilms (Mendez-Garcia et al., 2014).

### 2.3. Diversity of eukaryotes

The Tinto River constitutes the main reference for the diversity of eukaryotic microorganisms inhabiting AMD systems (Amaral Zettler et al., 2002, 2003; Aguilera et al., 2006, 2007; Amaral-Zettler, 2012). Including additional observations conducted at the Xiang Mountain (Hao et al., 2010), Königstein (Zirnstein et al., 2012), and Richmond (Baker et al., 2004, 2009; Aliaga Goltsman et al., 2014) mines, eukaryotes present in these environments primarily belong to the *Archaeplastida, SAR* (*Stramenophiles* + *Alveolates* + *Rhizaria*), *Excavata* (protists) and *Opisthokonta* taxa. AMD-related *Archaeplastida* include phototrophic red (*Rhodophyceae*) and green algae (mainly *Chlorophyta*); these organisms depend on sunlight and are present in open-air systems, such as the Tinto River (SW Spain). The *SAR* members present in acidic waters include diatoms (e.g., *Pinnularia*), ciliates such as *Oxytricha* and *Vorticella*, and cercomonads. Members of *Excavata* detected in AMDs include *Euglena* sp. (*Euglenozoa*), *Bodo saltans* (flagellate) and the *Heterolobosea* genera *Naegleria* and *Vahlkampfia*. *Rotifera* and *Fungi* (*Opisthokonta*) complete the general set of eukaryotes found in AMD emplacements. The *Ascomycota* and *Basidiomycota* fungi are the primary eukaryotic members in sub-surface low-pH biofilms thriving inside the Richmond mine (Iron Mountain) (Baker et al., 2004, 2009).

## 3. Metabolism and element conversion in AMD systems at the organismal level

**Figure 3:**
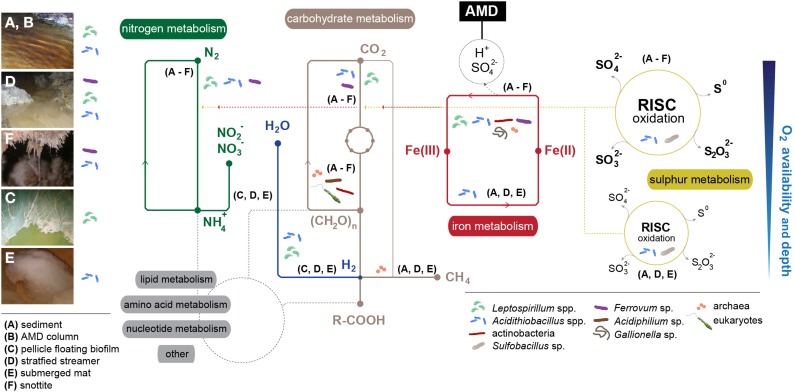
**Overview of the metabolic networks that govern adaptation to extreme conditions in AMD microbial communities by micro-environment (A–F)**. Main implicated taxa/groups are displayed. Dashed red-yellow lines represent chemical energy fueling carbon and nitrogen fixation. Dashed-gray lines display connections with lipid, amino acid, nucleotide and other metabolisms. Oxygen availability and depth refers only to nitrogen, carbohydrate, iron, and sulfur metabolisms. Main bacterial groups present in each environment have been included for a more intuitive understanding of the relationship between taxa and their spatial distribution.

The chemical species present in AMDs favor the establishment of a chemolithotrophy-based microbial community, whose general metabolism relies on the oxidation and reduction of iron and sulfur species (Figure 3). When macroscopic microbial growths develop, heterotrophy gains importance in the overall metabolism of the system. Based on the known metabolic capabilities of the autochthonous taxa, early AMD studies investigated the capacity of microbial species to metabolize iron, sulfur, nitrogen, carbon and oxygen; however, only a few initial studies described the sub-cycling of those elements in AMD systems. Notable examples include the various models proposed for iron and sulfur cycling, which are described below, by using biomolecular and cultivation-based methodologies, and more recently, by *omics*-based approaches.

### 3.1. Metabolism and element cycling inferred from taxonomy

#### 3.1.1. Tinto River

First models for iron and sulfur cycling were proposed for the Tinto River (Gonzalez-Toril et al., 2003; Rowe et al., 2007; Amils et al., 2011) (Figure 3). In one such model, microbes thriving in the sediments/water column of the Tinto River perform the oxidation of Fe(II) to Fe(III) and recovery of Fe(II) through Fe(III) reduction. Given that aerobic or microaerophilic conditions are defined by the sediment/water column transition area, the Fe(II) oxidation is conducted by iron oxidizers in the oxic zone, and the Fe(III) reduction step is performed by certain iron-sulfur oxidizers (e.g., acidithiobacilli) at low oxygen concentrations. Sulfur oxidation can occur uncoupled to ferric reduction, and sulfate reduction is driven by SRB in anoxic conditions. In phototrophic stratified streamers, the oxic metabolic activities involving sulfur/iron oxidation and oxygenic photosynthesis occur in the upper strata, whereas Fe(III) and sulfate reduction occur in the lower strata (Rowe et al., 2007). Several bacteria thriving in the Tinto River have the capacity to fix atmospheric CO_2_, but eukaryotic photosynthesis is proposed to be their main carbon source (Aguilera et al., 2006). Nitrogen intake might be mediated by the community members that can perform N_2_ fixation (e.g., *Acidithiobacillus ferrooxidans*, Valdes et al., 2008), and extra inorganic nitrogen could be taken up from other resources via common ammonium and nitrate transport systems.

#### 3.1.2. Cae Coch mine

The biogeochemistry of the Cae Coch AMD system was proposed by Kimura et al. (2011). By studying the microbiology of all of the strata within the mine, it was observed that, in the absence of light, all primary production is mediated by chemoautotrophs. The pyrite ore body can fuel chemolithotrophic metabolism, and ferrous iron might be the predominantly oxidized species (preferred over sulfur). In the Mynydd Parys (or Parys Mountain in English) underground lake, the iron could cycle as in the Cae Coch environment, but dissolved organic carbon (DOC) would also originate from residual wooden mine support structures (in addition to autotrophic iron- and sulfur-oxidizing bacteria), and would be utilized as an electron donor by heterotrophic bacteria and methanogenic archaea, thriving at higher depths within the system (Figures 3A–F).

#### 3.1.3. Königstein mine

In the snottites suspended from the Königstein uranium mine ceilings, bacteria are the first colonizers, and “*Ferrovum myxofaciens*” contributes primarily to the formation of the slimy structure (Figure 3F), with the subsequent emergence of a number of microscopic eukaryotes (Zirnstein et al., 2012). Flagellates, ciliates and rotifers act as primary/secondary consumers, and amoebae as secondary grazers. Fungi could participate in carbon recycling, acting as the main decomposers within the community.

### 3.2. Metabolism and element conversions inferred from *omics*-wide studies

Major limitations of using taxonomic data to reconstruct AMD metabolic networks are that they include uncertainties about the physiological features of the organisms and a high ratio of sequences that cannot be assigned to any known microorganism. Another major limitation is that the activity associated with some sequences might be unclear. Therefore, shotgun sequence data (community genomics or metagenomics) and the experimental measurement of protein levels (community proteomics or metaproteomics) may be better indicators of the element cycling in environmental samples. However, it is important to point out the limitations resulting from these experimental approaches, including that the activity associated with some sequences might be unclear or incorrectly assigned and that, due to regulatory effects, the experimental measurement of protein levels may not be synonymous with their activity. For all these reasons, experimental validation assays need to be conducted. Whatever the case, both *omics* techniques have been recently proven to efficiently contribute to the understanding of metabolism and element cycling in a number of AMD systems, which are detailed below.

#### 3.2.1. Carnoulès mine

The Carnoulès AMD system has the highest arsenic content of all reported acid drainages. The metaproteogenomics-based metabolic model for this system described by Bertin et al. (2011) proposes that five biological metabolic systems are responsible for the main chemical transformations within the arsenic-laden sediments. Sulfide and iron oxidation appear to fuel AMD formation and suggest the presence of iron/sulfur oxidizers. Nitrogen and carbon fixation are potentially performed by various members of the community (e.g., *Leptospirillum* spp.), while additional carbon intake into the system may depend upon photosynthetic *Euglena* thriving at the surface of the river. In the As-rich Carnoulès environment, arsenate-resistance mechanisms appear ubiquitously in different members of the community, and proteogenomic signatures for arsenite oxidation and methylation are also present (Bertin et al., 2011).

#### 3.2.2. Richmond mine

Tyson et al. (2004) and Ram et al. (2005) established the metaproteogenomic basis of the metabolic functioning of acidic biofilms in Iron Mountain (California). The high abundance of a few microbial types in this system permitted the reconstruction of complete genomes from the environment for the first time, as well as the elucidation of the relationships among community members. AMD floating biofilms thrive in the air-solution interface (oxic zone), with thicknesses ranging from 20 μm (early developmental stages) to 0.1 mm (late developmental stages) (Wilmes et al., 2009; Justice et al., 2012). At early developmental stages, thin biofilms are dominated by bacteria (mainly *Leptospirillum ferriphilum*-like and also“*Leptospirillum ferrodiazotrophum*”-like) and some archaea of the genus *Ferroplasma* (“type I” and “type II”). Possibly, heterotrophic archaea (including uncultured clades of the order *Thermoplasmatales*, primarily “A-plasma” and “G-plasma” variants), ARMAN and fungi (mainly *Acidomyces richmondensis*) are present at higher levels; however, these organisms exhibit low relative abundances in thick biofilms, dominated by *Leptospirillum ferriphilum*-like bacteria (“group II”)- at late developmental stages. The most complex study of carbon mobilization in AMD systems was performed by Justice et al. (2012). By using community genomics and proteomics, they proposed that *Leptospirillum* spp. are responsible for Fe(II) oxidation and carbon assimilation through *Leptospirillum ferriphilum*-like “group II” (Goltsman et al., 2009), and nitrogen fixation by “*Leptospirillum ferrodiazotrophum*”-like “group III” bacteria (Tyson et al., 2005) in the initial community, where biofilm formation would occur. Under anoxic conditions, the floating biofilm, which does not occupy the entire AMD column, is degraded in the sediment/drainage interface (sunken fraction). The authors proposed that air-solution biofilms use chemolithotrophy, and heterotrophy is the prevailing metabolic trait in sunken, actively degrading biofilms.

#### 3.2.3. Drei Kronen und Ehrt mine

The metabolic model proposed for the Drei Kronen und Ehrt pyrite mine snottites (Ziegler et al., 2013) is based on metagenomic data. The stalactite-like growths within the pyrite mine would originate from ferrous oxidation maintained by dominant *Leptospirillum* bacteria. The presence of *Leptospirillum* may be restricted to the oxic surface of snottites, and bacteria such as *Acidithiobacillus* spp. and “*Ferrovum*”-related species could be present in much smaller proportions, *Acidithiobacillus* spp. might perform both ferrous iron oxidation and reduction, depending on their location within the snottite, and “*Ferrovum*” spp. metabolism could rely on ferrous iron oxidation. The anoxic portion of the stalactite-like structure might be enriched with heterotrophic archaea (*Ferroplasma* spp. and uncultured representatives) (Figure 3F) (Ziegler et al., 2013).

#### 3.2.4. Los Rueldos mine

Mendez-Garcia et al. (2014) proposed a metaproteogenomics-based model for main microbial formations in a nearly stagnant AMD in NW Spain (Los Rueldos). In this model, dissolved oxygen is shown to be presumptively the primary force driving microbial diversity and associated metabolism. The communities thriving in the AMD-air interface are possibly more active in terms of iron, nitrogen and hydrogen usage, whereas the suboxic communities presumptively appear to have a major role in sulfur and carbon transformations. Differential metabolic capabilities shift rapidly in a small spatial gradient, and different species of the same genus co-habit discrete micro-environments defined mostly by the dissolved oxygen concentration.

## 4. Element cycling by *bacteria* and *archaea* in AMD sites at the biochemical level

### 4.1. Iron metabolism in AMD systems

**Figure 4:**
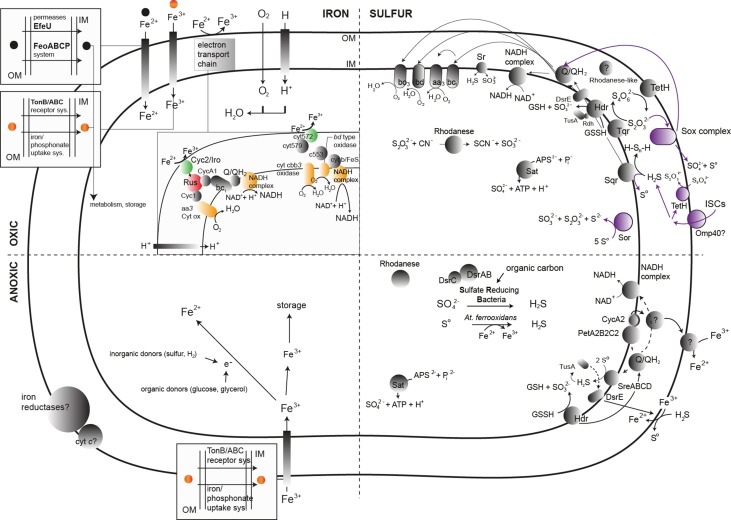
**General scheme of the main known biochemical transformations and the associated enzymes (represented by gray, green, red, purple, and yellow circles) implicated in the bacterial metabolism of iron and sulfur in AMD systems**. All possible sub-systems are represented in the quarters of each image. Color assignments are as follows: green, elements involved in the initial ferrous iron oxidation; red, key enzymes in the ferrous oxidation process; yellow, final electron acceptor in the ferrous iron oxidation cascade; purple, elements involved in the RISC metabolism in *Acidithiobacillus caldus*. The archaeal enzymes responsible for iron and sulfur transformations in AMD systems have been obviated in the figure due to the lack of accurate biochemical information relying on the culture of AMD-related archaea. Abbreviations not present in the main text: GSH, Glutathione; GSSG, Glutathione disulfide; PetAB2CC2, *petII* operon in *Acidithiobacillus ferrooxidans* ATCC 33020; Cyc2, Cytochrome *c* Cyc2; cyt572, Cytochrome *c* 572.

The primary biochemical transformation involving iron occurring in AMD systems is ferrous iron oxidation, implied by the dominance of iron-oxidizing bacteria as compared to that of species capable of ferric iron reduction (e.g., Tan et al., 2007). The related enzymes involve an electron transport chain where electrons are transferred from the reduced species to the first component, most likely an outer membrane cytochrome *c*, as in *Acidithiobacillus ferrooxidans* or *Leptospirillum ferrooxidans* (Figure 4). Other (periplasmic or membrane peripheral) proteins are only related to ferrous iron oxidation: the blue copper proteins rusticyanin, RusA (Amouric et al., 2011) and sulfocyanin in *Ferroplasma* spp. (Dopson et al., 2005), and the Iro (iron-oxidase) protein (Amouric et al., 2011). A super-complex from *Acidithiobacillus ferrooxidans* was characterized in detail and exhibited iron oxidase and oxygen reductase activities (Castelle et al., 2008; Roger et al., 2012). Under anoxic conditions, ferric iron could act as the terminal electron acceptor (Figure 4). The electron donors that are coupled to iron reduction could be inorganic (sulfur or hydrogen), in the case of chemolithotrophic acidophiles (*At. ferrooxidans*), or organic (glucose, glycerol), in the case of heterotrophic acidophiles (e.g., *Acidiphilium* spp.). The enzymatic system related to ferric iron reduction remains unknown, but evidence exists of that this process might involve iron reductases (Johnson and Hallberg, 2008).

### 4.2. Sulfur metabolism in AMD systems

Sulfur transformations are complex due to the variety of oxidation states adopted by this element. Sulfur and reduced inorganic sulfur compounds (RISCs) can accumulate in areas where pyrite and other sulfide minerals are oxidized by ferric iron (Schippers and Sand, 1999). Acidophilic sulfur-oxidizing bacteria can oxidize these RISCs, (polythionates such as thiosulfate and tetrathionate), along with sulfur and sulfide (Johnson and Hallberg, 2008; Dopson and Johnson, 2012). Reduced inorganic sulfur compounds constitute a more energetically favorable substrate than reduced iron. The process of RISC oxidation requires different enzymatic machineries depending on whether the sulfite is produced as an intermediate. As a tentative general scheme, during RISC oxidation through sulfite production, sulfide is oxidized by a sulfide/quinone oxidoreductase (Sqr), which transfers electrons to ubiquinone and generates sulfur (coding sequences found in genomes of *At. thiooxidans, At. ferrooxidans, At. caldus*, and *At. ferrivorans*), which could be oxidized to sulfite by the periplasmic sulfur dioxygenase (Rohwerder and Sand, 2003). Finally, sulfite would be oxidized to sulfate by the enzyme sulfite oxidoreductase or via the activity of the enzyme adenosine phosphosulfate (APS) reductase (encoded by *aprAB*). A similar coupling of enzymes is used for the oxidation of the polythionates (trithionate, tetrathionate and thiosulfate) by enzymes such as trithionate dehydrogenase (produces thiosulfate), and thiosulfate dehydrogenase (produces tetrathionate, found in *At. ferrooxidans* and *At. ferrivorans*). Tetrathionate hydrolysis is performed by tetrathionate hydrolase (TetH), and the produced thiosulfate is oxidized by a thiosulfate quinone oxidoreductase (DoxDA) (Quatrini et al., 2009). A role for the enzyme heterodisulfide reductase (Hdr) in the oxidative metabolism of RISCs in certain acidithiobacilli has been strongly supported by gene expression analysis during growth on different sulfur compounds. The Sox system (*At. thiooxidans, At. caldus*) oxidizes reduced sulfur compounds directly to sulfate without the formation of sulfite as an intermediate. This system is expressed from over 15 genes that encode various cytochromes and other proteins necessary for the oxidation of reduced sulfur compounds directly to sulfate. The presence of three terminal electron acceptors relates to RISC energy conservation: a *bd* ubiquinol oxidase (*cydAB, At. thiooxidans, At. ferrooxidans, At. caldus*), a *bo*_3_ oxidase (*cyoABCD*) and the *bc*_1_ complex, and cytochrome *c*_4_, encoded by the *petII* operon (*At. ferrooxidans, At. ferrivorans*) (Quatrini et al., 2005) (Figure 4).

Many of the RISC-metabolizing enzymes from archaea belonging to the order *Sulfolobales* have been characterized (Rohwerder and Sand, 2007). The major difference compared to other chemical transformations involving RISCs is that the key enzyme of the process is a sulfur oxygenase reductase (Sor), which catalyzes the disproportionation (simultaneous oxidation and reduction) of sulfur. The thermoacidophilic archaeal RISC oxidation-related proteins include the enzymes Sor, TetH and DoxDA (Auernik and Kelly, 2010).

RISC oxidation can be coupled to ferric iron reduction (Figure 4), a process that might involve Sqr (Pronk et al., 1990, 1991). Anaerobic growth via reduction of elemental sulfur has been described in four genera of acidophilic archaea: the thermoacidophilic crenarchaea *Acidianus, Stygiolobus*, and *Sulfurisphaera*, the moderately thermoacidophilic euryarchaeote *Thermoplasma*, and one bacterial strain of *Acidithiobacillus ferrooxidans* (Ohmura et al., 2002; Kucera et al., 2012; Osorio et al., 2013).

Reduction of sulfate, which ends with the formation of H_2_S (sulphidogenesis) (Figure 4), is performed by acidophilic or acid-tolerant SRB. This group plays a major role in the coupled biogeochemical cycling of sulfur and chalcophilic metal(loid)s (Moreau et al., 2010). The key enzymes of the process are the dissimilatory sulfite reductases (DSRs), which are multi-subunit enzymes that catalyze the six-electron reduction of sulfite to sulfide.

Sulfur assimilation (usually as sulfate) might be performed across the cellular membrane either via an ABC uptake system or the SulP sulfate permease in *Acidithiobacillus ferrooxidans* (Valdes et al., 2003). Sulfate is incorporated into the amino acids methionine and cysteine, iron-sulfur centers and other metabolites (Aguilar-Barajas et al., 2011). The production of cysteine occurs via the intermediates adenosine-5′-phosphosulfate (APS), sulfite and sulfide, and is encoded by the *cysJIHDNG* operon. A potential pathway for the sulfation of metabolites is the PAPS (3′-phosphoadenosine-5′-phosphosulfate) pathway, which might be encoded by two non-identical copies of *cysNC* (Valdes et al., 2003). APS reductase and sulfate adenylyl transferase (SAT) possibly act in the reverse direction compared to sulfate assimilation to produce ATP (Auernik et al., 2008).

### 4.3. Nitrogen metabolism in AMD systems

The main nitrogen transformations occurring at AMD sites include nitrogen fixation, ammonification, nitrification and denitrification.

In AMD systems, a few species can fix atmospheric N_2_: *Acidithiobacillus ferrooxidans* (Valdes et al., 2008), *Leptospirillum ferrooxidans* (Norris et al., 1995; Parro and Moreno-Paz, 2003), “*Leptospirillum ferrodiazotrophum*” (Tyson et al., 2005), and “*Ferrovum myxofaciens*” (Johnson et al., 2014) (Figures 3A–F). The metagenomic reconstruction of the main prokaryotic units in the Carnoulès AMD led to the observation that *Gallionella* and *Thiomonas* (undetermined species) might also be involved in nitrogen fixation (Bertin et al., 2011), but the *nif* operon has not been detected in the *Thiomonas* sp. 3As genome sequence (Arsene-Ploetze et al., 2010). Usually, those systems receive limited fixed carbon and nitrogen from external sources, and fixation of atmospheric CO_2_ and N_2_ (Tyson et al., 2005) by the microorganisms becomes crucial. Tyson et al. (2005) proposed that “*Leptospirillum ferrodiazotrophum*” is the key nitrogen fixer at pH levels below 1 in the acidic biofilm growing in the Richmond mine (Tyson et al., 2004). *Leptospirillum ferriphilum*-related bacteria (“group II”) retrieved from an acidic biofilm at the Richmond mine harbor various genes implicated in nitrogen fixation (Tyson et al., 2005). The recent proposition of *Leptospirillum* “group IV” (Goltsman et al., 2013) suggests the existence of genomic potential for carbon and nitrogen fixation in this group, along with the possibility of anaerobic growth using hydrogen as an electron donor.

N_2_ fixation is, with few exceptions, mediated by the Mo-Fe nitrogenase enzyme complex, whose activity is sensitive to the presence of oxygen. The enzymatic structural components are encoded by the *nif* operon (*nifHDKENX* genes). Genes flanking this operon (regulators, transporters, oxygen/redox sensors) can also be involved in nitrogen fixation (Parro and Moreno-Paz, 2004; Tyson et al., 2005).

Nitrogen in the form of ammonium can either be oxidized in the process by nitrification or directly assimilated into biomass (Figure 3). Nitrifiers utilize molecular oxygen as the terminal electron acceptor and are known to be sensitive to low pH (Hankinson and Schmidt, 1988; Jiang and Bakken, 1999) due to the lack and/or toxicity of substrates at those pH values. Therefore, the occurrence of nitrifiers at AMD sites strictly depends on the availability of O_2_. The two key enzymes involved in ammonium oxidation, ammonium monooxygenase and hydroxylamine oxidoreductase, are encoded by the *amoCAB* operon (AmoA contains the putative enzyme active site) and *hao* gene, respectively. *Leptospirillum* spp. of the so-called “group II” and “group III” contain the *amoA* gene in their metagenomes, but they lack the rest of the operon, which suggests that AmoA might be involved in other activities, such as methane oxidation or/and hydrocarbon degradation (Goltsman et al., 2009).

Nitrate or nitrite ions can be used as terminal electron acceptors in anoxic or low-oxygen conditions (denitrification). Baeseman et al. (2006) examined nitrogen metabolism in acidic, heavy metal-laden environments; however, in their microcosm study of sediments from acidic streams, it was suggested that denitrification occurs and may reduce the acidity. Xie et al. (2011) utilized the GeoChip to evaluate the functional gene diversity and metabolic potential of the microbial communities in AMD systems and inferred that denitrification is an active and integral part of the general system metabolism; nevertheless, additional analyses, including transcriptomic analysis and/or related proteins detection, as well as direct activity measurements would be needed to confirm the process is active.

Ammonification occurs via nitrate reduction followed by nitrite ammonification, and the enzymes involved include Nas, Nar, and Nap (nitrate reductases) and Nir and Nrf (nitrite reductases). In AMD systems, the putative presence of this activity in *Leptospirillum ferriphilum* “group II”-related bacteria was suggested based on the observation of a cytochrome *c* NapC/NirT family protein involved in respiratory nitrite ammonification. *Leptospirillum* spp. “group II” and “group III,” which were reported to contain genes for a nitrite/sulfite reductase, are required for assimilatory nitrite ammonification with the end-product, ammonium, being directly channeled into amino acid biosynthesis (Simon, 2002; Goltsman et al., 2009). Once ammonium enters the cells, it is assimilated by the glutamine synthase/glutamate synthase pathway (GS/GOGAT), which appears to be absent in *Leptospirillum*, where ammonia assimilation may occur via a GS pathway similar to that proposed for *At. ferrooxidans* (Tyson et al., 2005).

Most AMD community members do not fix nitrogen and must obtain it via ammonium uptake. For example, *Leptospirillum ferriphilum*-related bacteria (“group II”) have genes encoding three ammonium transporters clustered with the genes encoding nitrogen-regulatory PII proteins. This gene arrangement appears to be highly conserved and suggests that the regulatory proteins are related to ammonium uptake (Ninfa and Jiang, 2005; Goltsman et al., 2009). The *At. ferrooxidans* genome also contains genes predicted to be involved in ammonium uptake (*amt1, amt2*, and *amtB, AFE2916, AFE2911*, and *AFE1922*, respectively) and a gene encoding a protein that incorporates ammonium into glutamine (the *glnA*-homologous gene) (Valdes et al., 2008).

### 4.4. Carbon metabolism in AMD systems

As carbon sources are severely limited in acidic waters, microbial carbon cycling is of great interest in AMD ecosystems. Notably, some typical members of AMD communities contain genes that enable them to fix CO_2_ through different pathways. *Acidithiobacillus ferrooxidans, At. thiooxidans, At. caldus* (Valdes et al., 2008), “*Ferrovum myxofaciens*” (Johnson et al., 2014), *Sulfobacillus thermosulfidooxidans, Sb. acidophilus*, and *Acidimicrobium ferrooxidans* (Caldwell et al., 2007) may perform carbon fixation through the Calvin-Benson-Bassham (CBB) cycle (Figures 3A–F). Additionally, metagenomic signatures for CO_2_ fixation through the CBB cycle have also been associated with the *Gallionella* and *Thiomonas* genera in the Carnoulès lead/zinc mine (Bertin et al., 2011). Metagenomic analysis of the Richmond mine led to the prediction of that several *Leptospirillum*-related members in the community, such as “*Leptospirillum rubarum*,” “*L. ferrodiazotrophum*” (“group III”), and uncultured *Leptospirillum* spp. (“group IV”) (Tyson et al., 2004; Ram et al., 2005; Goltsman et al., 2009, 2013) (Table 2) may fix inorganic carbon through the reductive tricarboxylic acid (rTCA) cycle. Some *Ferroplasma* strains contain genetic determinants that may be involved in a reductive acetyl-coenzyme A pathway for carbon fixation (Tyson et al., 2004). Fixation of CO_2_ in acidophilic archaea may occur via the 3-hydroxypropionate/4-hydroxybutyrate cycle (genera *Acidianus* and *Metallosphaera*) or the reductive acetyl-CoA pathway (not yet demonstrated).

In AMD systems, heterotrophy and autotrophy can be either obligate or facultative. Obligate heterotrophs belong to the bacterial class *Alphaproteobacteria* (*Acidiphilium* spp. with the exception of *A. acidophilum, Acidocella, Acidomonas, Acidisphaera*, and *Acidicaldus*), and the phyla *Firmicutes* (*Alicyclobacillus*, facultative but “heterotrophically inclined”) and *Actinobacteria* (*Ferrimicrobium, Ferrithrix*). Facultative metabolism can be observed in the bacteria *Acidiphilium acidophilum, Sulfobacillus, Alicyclobacillus* and *Acidimicrobium ferrooxidans*. Obligate heterotrophic archaea in AMD biotopes include the genera *Thermoplasma*, some *Ferroplasma* spp., and some *Acidiplasma* spp. (*Euryarchaeota*). Crenarchaeal representatives, more common in high-temperature volcanic environments, include obligate heterothrophic members of the genera *Sulfolobus* and facultative heterotrophic members of *Metallosphaera, Sulfurococcus, Acidianus, Sulfurisphaera*, and *Acidilobus* (Johnson and Hallberg, 2008).

The most complete study regarding carbon mobilization in the model AMD system of the Richmond mine has been conducted by Justice et al. (2012) (Section 3.2.2). In such one model, carbon transformations involving a floating biofilm and its sunken fraction, as well as the responsible microbial groups, are described.

#### 4.4.1. Methane production in AMD environments

Macroscopic growths and the subterranean lake present in the Mynydd Parys mine contained taxonomic markers (SSU rRNA sequences) similar to those from methanogens. Johnson (2012) observed that the ecological impact of these “presumptive” methanogens was unclear. Sanz et al. (2011) observed that methanogenesis exists in enrichment cultures from sediments of the Tinto River. In a survey of the archaeal diversity in arsenic-rich creek sediments of the Carnoulès mine, Volant et al. (2012) observed microorganisms related to methanogenic archaea that included, among other species, *Methanomassiliicoccus luminyensis*. Archaea related to the same species were also detected in low-pH suboxic macroscopic growths from the Los Rueldos mine (Mendez-Garcia et al., 2014). However, no methanogens capable of growth at pH levels below 3, or enzymes pivotal for methanogenesis, have been isolated/detected in AMD systems (Johnson, 2012).

### 4.5. Hydrogen metabolism in AMD habitats

Hydrogen (H_2_) is a common product that is generated during microbial metabolic transformations and fermentations, and a number of chemolithotrophs can use it in energy metabolism.

Bacteria and archaea that perform hydrogen oxidation differ in their electron acceptors, e.g., nitrate, oxygen, sulfate, ferric iron, and others. The key enzymes in the process are hydrogenases, which catalyze the reversible oxidation reaction of H_2_ to protons. These enzymes are highly versatile, can use a variety of substrates, and may act as electron mediators during hydrogen generation; therefore, hydrogenases have various functions that are not directly linked to H_2_ transformations, but rather to electron transport.

The importance of hydrogen as an electron donor for acidophiles is not well understood. The presence of hydrogenase genes in several sequenced genomes of acidophiles (e.g., *Acidithiobacillus ferrooxidans*, Valdes et al., 2008) and the observation that at least some known species of acidophiles can grow autotrophically on hydrogen in acidic liquors (Drobner et al., 1990) indicate the importance of hydrogen as an electron donor for these bacteria (Johnson, 2012). Furthermore, genes encoding hydrogenases were also observed in the metagenome of Carnoulès sediments (Bertin et al., 2011), in *Leptospirillum* spp. “group IV” (Goltsman et al., 2013), and hydrogenases were identified in the metaproteome of Los Rueldos macroscopic growths (Mendez-Garcia et al., 2014).

### 4.6. Element cycling by archaea from the filterable fraction in AMD habitats

The filterable archaeal fractions of macroscopic growths in low-pH sub-surface niches are suggested to have a heterotrophic lifestyle in both the presence (Baker et al., 2010) or absence (Ziegler et al., 2013) of oxygen. Their contribution to the gross carbon turnover in community metabolism is proposed to be low (Baker et al., 2010). Their genomic features (small genome sizes, short genes, split, and/or overlapping genes) are shared by host-associated/symbiotic microbes, suggesting that the ARMAN may depend upon other community members for some fraction of their resources and metabolites. A physical relationship between some pleomorphic cells, which are believed to be members of the order *Thermoplasmatales*, and smaller counterparts, which are suggested to be ARMAN cells, has also been reported (Comolli et al., 2009). This observation could explain why archaea from the filterable fraction would thrive in macroscopic growths developing independently from the oxygen influence in some environments. The fact that enrichment cultures or isolates have not been produced thus far limits the understanding of the biology of these curious, recently discovered microorganisms.

### 4.7. Implications of eukaryotes in the element cycling of AMD environments

Protozoa thriving in AMD systems may feed upon prokaryotic producers to sustain their heterotrophic metabolism, acting as primary (ciliates, flagellates and rotifers), or secondary (ciliates, rotifers and amoebae) consumers within the community. Low-pH conditions favor the development of fungi, which act as decomposers and contribute to carbon recycling (Figure 3). When present, microscopic algae act as primary producers in open-air AMD flows (e.g., in the Tinto River). Although certain eukaryotes are able to thrive under acidic conditions, they are mainly neutrophilic, and their numbers are higher in less extreme pH conditions (Lopez-Archilla et al., 2001; Amaral Zettler et al., 2002; Sabater et al., 2003; Aguilera et al., 2006; Baker et al., 2006). However, microscope observations have revealed that the eukaryotic (similarly to the prokaryotic) community is primarily distributed in diverse biofilms. This adaptation may protect their members from the extreme external conditions (Aguilera et al., 2006), thus allowing them to significantly contribute to the overall metabolism in AMD systems. Eukaryotes associations with other bacterial and archaeal species might have an impact in the metabolism of certain (bio)chemicals (Aguilera et al., 2006). For instance, in a biofilm, algae can perform photosynthesis by taking up CO_2_ that is excreted by heterotrophic bacteria or archaea; in turn, the algae provide organic substrates for these microorganisms (Aguilera et al., 2006).

The extent of eukaryotic roles is still unclear for a number of reasons and awaits further studies. First, it is difficult to identify eukaryotic genomic sequences through direct DNA sequencing due to their low cell numbers compared with archaea and bacteria in the majority of AMD habitats. Second, identified species are in some cases extremely divergent from any organism that have been cultured or sequenced (Baker et al., 2006). Therefore, this topic represents an attractive opportunity for research.

## 5. Concluding remarks

AMD systems are represented in a variety of environments in which biological niches are shaped by certain environmental conditions, with low pH being the most important. The differences in microbial diversity among AMD-impacted environments are present at low taxonomic levels and reveal the existence of environment-driven fine-tuning of the microbial populations. The autochthonous genetic pool might have originated natively, or might have entered the environment through unknown dispersal strategies; in this sense, comparative ecology studies over a broader selection of environments (e.g., hot springs) would extend our understanding about the origin and ecological succession of the microbial communities in acidic environments. Additionally, the inference of the role of many detected members belonging to different taxonomic groups (e.g., Candidate division TM7) is still unclear, and expansion of this knowledge would result in a more accurate depiction of the microbial ecology in AMD systems.

Acid mine drainage triggered by anthropogenic mining activities represents a significant environmental problem. The ecosystems emerging as a result of AMD are extremely acidic and metal-rich, and the associated microbiome is restricted almost entirely to only a few most abundant taxa of specialized bacteria and archaea. These habitats contain unique microorganisms and novel metabolic functions selected to cope with the harsh conditions, some of which, as well as the metabolites they produce, might be of biotechnological relevance. The presence of a filterable archaeal fraction in microbial communities of the AMD necessitates further genomic diversity studies in environments of a similar nature and stimulates the debates regarding the origins of life. Microbial ecology focuses on mechanisms of functioning biological systems and their interaction with the abiotic environment (lithosphere). Therefore, to obtain a general view on how biological systems function, the approaches should be very inclusive.

Further efforts should be conducted to more in-depth sequencing and cultivation studies. Only a detailed survey of complete genomes, whose production from metagenomes and single-cells is still technically challenging, will facilitate an accurate prediction of microbial metabolism; in turn, the experimental investigation of physiological traits in pure cultures brings us to a more comprehensive understanding of metabolism of the members of AMD communities and to a full assessment of their biotechnological prospects. Obviously, introducing new isolation approaches to bypass all the difficulties in the cultivation of AMD-related microorganisms (differential growth rates among fast and slow-growing prokaryotes; specific, often unknown, media requirements; determination of oligotrophic conditions for growth) will improve our current knowledge on archaeal and bacterial lineages within AMD habitats, whereas the main attention should be paid to cellular communications and any form of interactions between microbial partners important in natural environments.

Finally, a greater effort to culture the individual members of AMD communities and to establish stable low-complexity enrichment cultures to include co-cultures of archaea from the filterable fraction will facilitate new and exciting discoveries in coming years.

### Conflict of interest statement

The authors declare that the research was conducted in the absence of any commercial or financial relationships that could be construed as a potential conflict of interest.
